# Local and Transient Changes of Sleep Spindle Density During Series of Prefrontal Repetitive Transcranial Magnetic Stimulation in Patients With a Major Depressive Episode

**DOI:** 10.3389/fnhum.2021.738605

**Published:** 2022-01-06

**Authors:** Takuji Izuno, Takashi Saeki, Nobuhide Hirai, Takuya Yoshiike, Masataka Sunagawa, Motoaki Nakamura

**Affiliations:** ^1^Laboratory of Neuromodulation, Kanagawa Psychiatric Center, Yokohama, Japan; ^2^Department of Physiology, Showa University School of Medicine, Showa University, Tokyo, Japan; ^3^Department of Psychiatry, Yokohama City University School of Medicine, Yokohama, Japan; ^4^Department of Neuropsychiatry, Graduate School of Medicine, Tokyo Medical and Dental University, Tokyo, Japan; ^5^Department of Sleep-Wake Disorders, National Institute of Mental Health, National Center of Neurology and Psychiatry, Tokyo, Japan; ^6^Medical Institute of Developmental Disabilities Research, Showa University, Tokyo, Japan

**Keywords:** repetitive transcranial magnetic stimulation, sleep spindle, slow wave sleep, neuroplasticity, sleep disturbance, major depression

## Abstract

The neuromodulatory effects of brain stimulation therapies notably involving repetitive transcranial magnetic stimulation (rTMS) on nocturnal sleep, which is critically disturbed in major depression and other neuropsychiatric disorders, remain largely undetermined. We have previously reported in major depression patients that prefrontal rTMS sessions enhanced their slow wave activity (SWA) power, but not their sigma power which is related to sleep spindle activity, for electrodes located nearby the stimulation site. In the present study, we focused on measuring the spindle density to investigate cumulative effects of prefrontal rTMS sessions on the sleep spindle activity. Fourteen male inpatients diagnosed with medication-resistant unipolar or bipolar depression were recruited and subjected to 10 daily rTMS sessions targeting the left dorsolateral prefrontal cortex (DLPFC). All-night polysomnography (PSG) data was acquired at four time points: Adaptation, Baseline, Post-1 (follow-up after the fifth rTMS session), and Post-2 (follow-up after the tenth rTMS session). Clinical and cognitive evaluations were longitudinally performed at Baseline, Post-1, and Post-2 time points to explore associations with the spindle density changes. The PSG data from 12 of 14 patients was analyzed to identify sleep spindles across the sleep stages II–IV at four electrode sites: F3 (frontal spindle near the stimulation site), F4 (contralateral homologous frontal region), P3 (parietal spindle in the hemisphere ipsilateral to the stimulation site), and P4 (contralateral parietal region). Statistical analysis by two-way ANOVA revealed that spindle density at F3 increased at Post-1 but decreased at Post-2 time points. Moreover, the local and transient increase of spindle density at F3 was associated with the previously reported SWA power increase at F3, possibly reflecting a shared mechanism of thalamocortical synchronization locally enhanced by diurnal prefrontal rTMS sessions. Clinical and cognitive correlations were not observed in this dataset. These findings suggest that diurnal rTMS sessions transiently modulate nocturnal sleep spindle activity at the stimulation site, although clinical and cognitive effects of the local changes warrant further investigation.

## Introduction

Sleep-related complaints that are most frequently accompanied by a depressive episode are not only symptoms but also risk factors for mood disorders (Baglioni et al., 2011). During a major depressive episode, insomnia and hypersomnia are, respectively, reported in approximately 80% and 15–35% of patients (Steiger and Pawlowski, 2019). Even in partial or complete remission phase, 43% of patients suffer from insomnia, while residual insomnia is a risk factor for non-remission (Yoshiike et al., 2017, 2020). Sleep abnormalities observed in major depression are characterized by sleep fragmentation, disinhibition of rapid eye movement (REM) sleep, and inhibition of non-REM (NREM) sleep (Benca et al., 1997; Armitage, 2007). More specifically, sleep fragmentation comprises prolonged sleep latency, frequently interrupted sleep, and early-morning awakening. The disinhibition of REM sleep involves shortening of REM latency, increased REM density during the first REM period, and prolonged REM periods. Finally, inhibition of NREM sleep implicates decreased stage II and slow wave sleep (stages III and IV), which can result in excessive daytime sleepiness.

Most antidepressants inhibit the abnormally enhanced REM sleep in depressive patients (Dunleavy et al., 1972; Kluge et al., 2007) but also normal REM sleep in healthy volunteers (von Bardeleben et al., 1989). This pharmacological effect on REM sleep suppression may underlie the therapeutic actions of antidepressants, considering the reported antidepressant effects of selective REM sleep deprivation (Vogel et al., 1975). Meanwhile, it was also shown that some antidepressants increase the delta band power during NREM sleep (Chen, 1979; Kluge et al., 2007).

Unlike medication, brain stimulation therapies for depression such as electroconvulsive therapy (ECT) and repetitive transcranial magnetic stimulation (rTMS) do not affect the sleeping brain directly. To date, little attention has been paid about the neuromodulatory effects of diurnal brain stimulation therapies on nocturnal sleep. It has been reported that ECT administration in medicated patients with major depression suppressed REM density, especially for the first REM period, and increased NREM sleep, especially slow wave sleep (Hoffmann et al., 1985; Goder et al., 2016). Although these findings are intriguing, it remains unclear whether these changes of sleep characteristics are due to primary or secondary effects of a series of ECT sessions. It should be underscored here that any localized changes of NREM or REM sleep were not reported in previous sleep studies evaluating the effects of antidepressants or ECT.

As compared with ECT, rTMS performed with a figure 8-shaped coil achieves a focal neurostimulation with a spatial resolution of 5–10 mm over superficial cerebral cortices (Jalinous, 1991). Since the TMS-induced action potentials spread *via* commissural, association, and projection fibers, rTMS is highly selective to their corresponding neuronal circuitries (Pascual-Leone et al., 2011). Considering this selectivity, the reciprocal thalamocortical circuitry, underlying the synchronized nature of NREM sleep, may be locally affected by rTMS. A seminal work by Huber et al. (2007) demonstrated in healthy volunteers that high-frequency rTMS over the motor cortex induced cortical potentiation in the ipsilateral premotor cortex as an immediate after effect, resulting in local enhancement of slow wave activity (SWA) in the same region during subsequent sleep.

Inspired by their work, we have previously reported that high-frequency rTMS sessions over the left dorsolateral prefrontal cortex (DLPFC) in patients with major depression locally enhanced SWA power around the stimulation site during nocturnal sleep (Saeki et al., 2013). However, the sigma (11–15 Hz) power indicative of sleep spindle activity during NREM sleep did not change significantly over time. We postulated that not only SWA power but also sigma power may increase following the rTMS sessions, because the thalamocortical synchronization during NREM sleep involves thalamocortical neurons as well as thalamic reticular neurons serving as a spindle oscillator (Steriade et al., 1993). In the present study, we focused on the spindle density, the occurrence rate of sleep spindles, rather than the sigma power, to provide further insights into a different aspect of the sleep spindle activity.

To our knowledge, no study has reported yet the rTMS induced alteration of sleep spindle activity in patients with major depression. All-night natural sleep EEG recordings were carried out four times throughout the present study protocol, including 10 sequential rTMS sessions over the left DLPFC across 2 weeks, allowing to evaluate their cumulative effects on the sleep spindle activity. We hypothesized that the prefrontal sleep spindle density can be locally enhanced by rTMS sessions, as it shares an underlying mechanism with the previously reported enhancement of prefrontal SWA power.

## Materials and Methods

### Study Participants

Inpatients with a DSM-IV-TR (APA, 2000) diagnostic of monopolar or bipolar depression were recruited for this open-label study. It should be noted here that participants and their entire dataset are completely identical to our previous study (Saeki et al., 2013). Participants with major sleep disorders (AASM, 2005) such as sleep apnea, circadian rhythm disorder, parasomnia, narcolepsy, or other primary sleep disorders were excluded from this study. Participants with a history of neurological disorders, epilepsy, head trauma, or substance abuse were similarly excluded. Those meeting contraindications for TMS such as intracranial ferromagnetic implants and a cardiac pacemaker were also excluded. According to these criteria, fourteen male inpatients were recruited at the Kanagawa Psychiatric Center, Yokohama, Japan. Only male patients were recruited considering that only male staff was available to monitor the patients during all-night PSG recordings, performed in an EEG room within a different building from their inpatients ward. All participants suffered from insomnia related to major depressive episodes as defined by the DSM-IV-TR. The participants underwent 10 daily rTMS sessions, clinical and neuropsychological evaluation, and all-night polysomnography (PSG) recordings for four nights including an adaptation night.

This study was approved by the ethics committee of the Kanagawa Psychiatric Center (KPC201107, UMIN000001185). Prior to study participation, every participant provided a written informed consent based on the Declaration of Helsinki.

Due to significant recording artifacts caused by electrode instability and oversensing of electromyographic activity, PSG data from two participants was excluded from the analysis. Consequently, data from 12 out of the 14 patients (mean age of 47.5 ± 6.3 years) was analyzed, including seven patients with unipolar depression and five patients with bipolar depression. Also, one patient could not complete the fourth night of PSG recording for personal reasons. For this patient, data for the first three nights of PSG recording are included in the present analyses.

### Experimental Design

**FIGURE 1:**
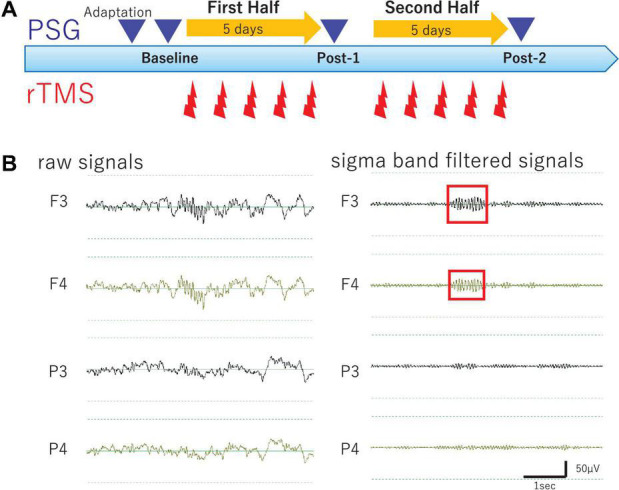
Study Protocol and Raw EEG data. **(A)** Experimental protocol. Diagram illustrates the experimental protocol of the present study. To evaluate cumulative effects of diurnal rTMS sessions on the nocturnal sleep, PSG recordings were performed over four nights: Adaptation, Baseline, Post-1 (after the fifth rTMS session), and Post-2 (after the tenth rTMS session). In each rTMS session over left DLPFC, 1,000 pulses were delivered as 25 trains of 2 s at 20 Hz with an intertrain interval of 28 s. **(B)** Raw EEG data of all night polysomnography recordings. An example of raw EEG data was illustrated from four monopolar derivations of F3, F4, P3, and P4 during NREM Stage II sleep. Sigma band filtered signals were illustrated at the right.

Methodological details of the present study are described elsewhere (Saeki et al., 2013). Briefly, in the present open-label study, participants underwent 10 daily rTMS sessions and four all-night PSG recordings, as well as longitudinal clinical and cognitive evaluations during hospitalization. An outline of the experimental protocol is illustrated in Figure 1A. Of note, medication protocols were kept unchanged throughout the study period in order to minimize potential effects of medication changes on PSG and clinical symptoms.

### Repetitive Transcranial Magnetic Stimulation Procedure

In a daily rTMS session, 1,000 pulses were delivered as 25 trains of 40 pulses at 20 Hz with an intertrain interval of 28 s. According to the participants’ tolerability, the intensity of rTMS was adjusted to represent between 80 and 110% of the individual resting motor threshold. During a 2-week period of hospitalization, 10 daily rTMS sessions were administered corresponding to 10,000 pulses in total received for each participant. Of note, every rTMS session was performed between 9 am and noon, allowing to evaluate the cumulative effects rather than the acute after effect of rTMS sessions on the PSG recording performed later during the same day.

The above-described high-frequency rTMS was delivered to the left DLPFC, using a Magstim Rapid system (Magstim Company Ltd., United Kingdom) with an air-cooled figure 8-shaped coil (70 mm; Magstim). To deliver precise and consistent stimulation, the TMS coil center location, where is at the middle third of the middle frontal gyrus, was determined by a real-time ultrasound-based navigation system (zebris Medical GmbH, Germany; BrainVoyager TMS Neuronavigator, Brain Innovation, Netherlands), combined with a 3D reconstruction image from the individual brain magnetic resonance imaging (MRI) data. During a rTMS session, the TMS coil was held by hand with an appropriate pitch and roll angle tangentially to the scalp and with a yaw angle parallel to the sagittal plane.

### Polysomnography, Clinical, and Cognitive Data Acquisition

Each participant underwent PSG recordings during natural sleep from 9 pm to 6 am to acquire the following four PSG data; PSG data of adaptation night (Adaptation), baseline PSG data before the rTMS session (Baseline), follow-up PSG data after the fifth rTMS session (Post-1), and follow-up PSG data after the tenth rTMS session (Post-2). All-night PSG recordings were performed using a digital amplifier (Grass Technologies, West Warwick, RI), an elastic cap of 19 electrodes of the international 10–20 system for electroencephalography (EEG) measurement, and additional electrodes for electromyography (EMG) as well as electrooculography (EOG) measurements. During recording, skin-electrode impedance was kept below 10 kΩ. Band-pass filter (0.3–70 Hz) was applied to raw EEG signals with sampling rate of 400 Hz.

For clinical/cognitive evaluation, the 24-item Hamilton Depression Rating Scale (HAM-D 24) (Hamilton, 1960; Mazure et al., 1986), Beck Depression Inventory (BDI) (Beck et al., 1961), Wisconsin Card Sorting Test (WCST) (Grant and Berg, 1948), and word fluency test (Benton and Hamsher, 1976) were longitudinally assessed to explore possible changes following rTMS sessions and their correlations with PSG changes. Also, the five principal sleep disturbance components of the HAM-D (Milak et al., 2005) and the Kwansei Gakuin Sleepiness Scale (KSS) (Ishihara et al., 1982) were longitudinally evaluated to assess subjective sleep-related complaints of patients with major depressive episodes.

### All-Night Sleep Electroencephalography Data Analysis

Electroencephalography signals of the 19 monopolar derivations, chin EMG, and EOG were displayed on a computer screen using the sleep EEG browser/analyzer software (AWA) originally developed by one of the authors (NH). Every 30-s epoch with prominent artifacts was visually removed from the analysis. Firstly, sleep stage scoring was visually conducted for every 30-s epoch according to the standard Rechtschaffen-Kales criteria (Rechtschaffen and Kales, 1968). Secondly, spectral power values (μV^2^) of the delta (0.5–5 Hz) and sigma band (11–15 Hz) were calculated as power spectrum density for every 30-s epoch using Discrete Fourier Transform implemented in AWA (see Saeki et al., 2013 for detail). Thirdly, sleep spindles were visually identified by a single rater (TI) to investigate the sleep spindle density in NREM Sleep Stages II–IV. Finally, the amplitude, which is an average potential based on root-mean-square values, and the duration of each bandpass filtered (11–15 Hz) spindle waveform were measured using AWA to investigate morphological changes of spindle waveform over time. Of particular note, the rater was completely blinded to the data profiles such as identification of participants and time points of the PSG recordings. To this end, all PSG data files were completely renamed using a random number table exclusively for this analysis by a database manager (SO) and their header information including the subject profiles were totally excluded. The correspondence table of the PSG data file name linked to subject profile was password-protected by the database manager and thus inaccessible to everyone else until the analysis completion.

Sleep spindles were defined as waxing and waning waveform of sigma band oscillations lasting for 0.5–2.0 s during Stages II–IV, including two kinds of parietal and frontal spindles (Nakamura et al., 2003). Sleep spindle density was calculated as total number of sleep spindles per minute during Stages II–IV. In order to investigate a locality of rTMS-induced changes of the sleep spindle density over time, the following four electrode sites were selected for analysis: F3 (frontal spindle near the stimulation site of rTMS), F4 (contralateral homologous frontal region), P3 (parietal spindle in the hemisphere ipsilateral to the stimulation site), and P4 (contralateral parietal region). Spindle waveforms were visually identified based on the above definition at each electrode in Stages II–IV, after EEG signal was processed by the sigma band-pass filter (11–15 Hz). Intra-rater reliability of sleep spindle detection was evaluated in order to confirm its methodological consistency. To this end, an independent dataset was created by a database manager (SO). In this dataset, PSG data were renamed so that the subject name and test timing information would not be known to the rater (TI). The randomly selected eight PSG recordings were duplicated with different names as two distinct data. For each dataset, the sleep spindle waves of the F3, F4, P3, and P4 electrodes were visually identified. Thus, this dataset provided 32 pairs of spindle density values. The intra-rater reliability of sleep spindle density in the 32 pairs was 0.95 as assessed using Cronbach’s alpha, which was reasonably high. Additionally, the validity of the visually detected sleep spindles was estimated by determining the correlation between the averaged spindle density and the automatically calculated sigma power at corresponding electrode sites. Observed significant cross-sectional correlations (*r* = 0.576, *p* < 0.0001, *N* = 35) between the spindle density and sigma power density may indirectly validate the use of the present manual method to assess sleep spindle density.

### Statistical Analysis

Using SPSS software (version 23.0; SPSS Inc., Chicago, IL), the averaged sleep spindle densities at the selected four electrode sites (F3, F4, P3, and P4) were analyzed by two-way repeated-measures analysis of variance (ANOVA) with “time” and “electrode site” as the within-subject factors. When main effects of time and/or time-by-site interaction were statistically significant in the two-way ANOVA model, one-way ANOVAs and subsequent paired *t*-tests were performed as *post hoc* tests. *Bonferroni* correction was applied to each *post hoc* test, setting alpha levels of 0.0125 for one-way ANOVAs and 0.0167 for paired *t*-tests. Physiological correlations among spindle densities and sigma/delta powers and clinical/cognitive correlations with spindle densities were analyzed by Pearson’s or Spearman’s correlation analyses, depending on the Shapiro-Wilk normality test. The alpha level of the exploratory correlation analyses was set at 0.05 without any correction for multivariate correlations.

## Results

The two-way ANOVA analysis of spindle density with the within-subject factors “time” (Baseline, Post-1, and Post-2) and “site” (for the visually analyzed four electrode sites; F3, F4, P3, and P4) revealed a significant main effect of site [*F*_(3_,_30)_ = 5.542, *p* = 0.004] and also a significant time-by-site interaction [*F*_(6_,_60)_ = 3.193, *p* = 0.009], while a main effect of time [*F*_(2_,_20)_ = 2.295, *p* = 0.127] was not statistically significant. As reflected by the significant main effect of site, parietal spindles were more frequently observed than frontal spindles, which is compatible with the findings form previous studies (Nakamura et al., 2003).

**FIGURE 2:**
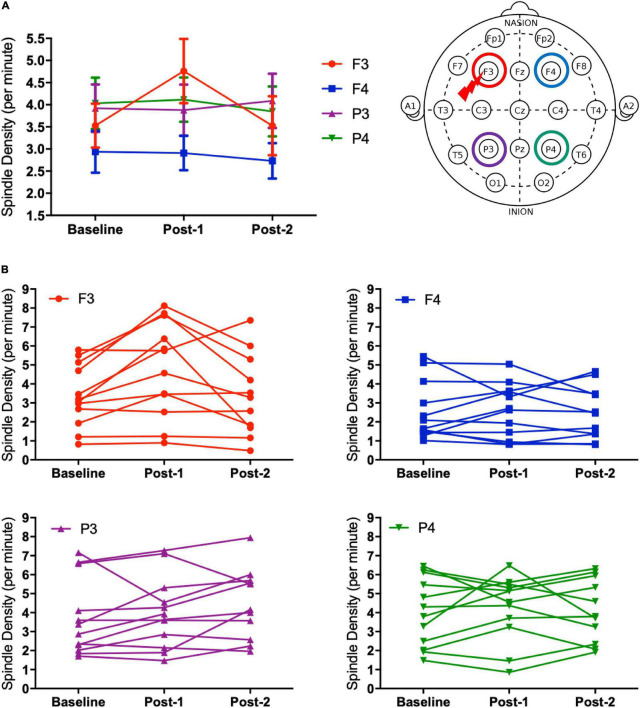
Spindle density changes over time. **(A)** The graph at the left shows sleep spindle density, which is the number of spindles recorded per minute during NREM sleep Stages II–IV, from four derivations of F3, F4, P3, and P4. As illustrated at the right, the F3 electrode is closer to the stimulation site of rTMS sessions. Spindle density at F3 increased from Baseline to Post-1 (*P* = 0.008) and then decreased from Post-1 to Post-2 (*P* = 0.044). **(B)** Inter-individual differences in spindle density change over time. Individual data was presented in four figures of F3, F4, P3, and P4 electrodes.

Considering that the time-by-site interaction was significant, *post hoc* one-way ANOVA was performed for each electrode site. The main effect of time was significant at F3 electrode [*F*_(2_,_20)_ = 5.519, *p* = 0.012] but not significant at the three other sites. Subsequently, *post-hoc* paired-*t* tests at the F3 electrode showed that the spindle density increased significantly between Baseline and Post-1 (*t*_11_ = –3.265, *p* = 0.008, Cohen’s *d* = 0.58) and exhibited a decreasing trend between Post-1 and Post-2 *t*_11_ = 2.304, *p* = 0.044, Cohen’s *d* = 0.53 (Table 2 and Figure 2). No significant difference in spindle density at F3 was found between the Baseline and Post-2 time points. Figure 1B illustrates the longitudinal changes of sleep spindle density measured at each electrode site, emphasizing the sleep spindle density increase for the F3 electrode, located nearby the stimulation site in the serial rTMS sessions. Descriptive statistics values are summarized in Table 1 along with the percentages of sleep stages across the total sleep time. At Post-1, density of the frontal spindles from F3 electrode increased to reach the same level as the parietal spindles from P3/P4 electrodes before returning to the level of frontal spindles at Post-2.

**TABLE 1:** Sleep variables.

					*P*-value (paired *T* test)			
	Adaptation	Baseline	Post-1	Post-2	Adaptation vs. Baseline	Baseline vs. Post-1	Baseline vs. Post-2	Post-1 vs. Post-2
TST (SD) [minutes]	462.5 (48.6)	469.3 (53.6)	449.5 (63.2)	452.4 (42.8)	0.664	0.308	0.367	0.517
Stage I (SD) [minutes]	100.7 (41.8)	101.5 (43.0)	85.8 (34.5)	95.2 (25.6)	0.958	0.112	0.877	0.135
Stage II–IV (SD) [minutes]	268.4 (68.4)	259.9 (73.6)	270.5 (71.1)	273.9 (42.7)	0.345	0.359	0.344	0.814
REM (SD) [minutes]	96.1 (39.2)	111.8 (56.7)	96.1 (37.3)	83.3 (22.2)	0.227	0.410	0.106	0.246
WASO (SD) [times]	3.3 (2.6)	4.1 (3.8)	2.4 (3.2)	3.1 (2.4)	0.399	0.122	0.190	0.111
WASO (SD) [minutes]	11.3 (9.6)	13.2 (12.5)	11.0 (14.8)	23.3 (24.3)	0.563	0.591	0.156	0.103
%TST								
Stage I (SD) [%]	22.4 (8.9)	22.3 (9.5)	20.2 (8.5)	21.5 (5.4)	0.984	0.484	0.805	0.399
Stage II–IV (SD) [%]	59.0 (12.3)	55.6 (13.8)	60.9 (10.7)	61.8 (7.2)	0.231	0.033*	0.191	0.904
REM (SD) [%]	21.2 (8.3)	24.0 (12.8)	21.8 (7.4)	18.7 (4.5)	0.287	0.554	0.144	0.132

TST, Total Sleep Time; SD, Standard Deviation; REM, Rapid Eye Movement; WASO, Wake After Sleep Onset; *: P < 0.05.

**TABLE 2:** Descriptive data.

	Baseline	Post-1	Post-2	*P*-value (paired *T* tests)		
				Baseline vs. Post-1	Baseline vs. Post-2	Post-1 vs. Post-2
total time of NREM (Stage II–IV) (SD) [minutes]	259.9 (73.6)	270.5 (71.1)	273.9 (42.7)	0.359	0.344	0.814
F3 total number of spindle (SD) [number]	874.9 (506.5)	1277.1 (747.9)	957.4 (620.6)	0.012*	0.390	0.077
F3 spindle density (SD) [number/time]	3.5 (1.7)	4.8 (2.5)	3.5 (2.2)	0.008*	0.961	0.044
F4 total number of spindle (SD) [number]	723.5 (429.1)	774.6 (406.8)	756.7 (404.3)	0.446	0.763	0.650
F4 spindle density (SD) [number/time]	2.9 (1.6)	2.9 (1.3)	2.7 (1.3)	0.905	0.479	0.183
P3 total number of spindle (SD) [number]	1003.3 (582.6)	1058.5 (580.4)	1134.4 (595.8)	0.538	0.192	0.258
P3 spindle density (SD) [number/time]	3.9 (1.9)	3.9 (2.0)	4.1 (2.0)	0.857	0.441	0.175
P4 total number of spindle (SD) [number]	1023.8 (554.4)	1126.3 (500.4)	1068.2 (543.6)	0.227	0.431	0.660
P4 spindle density (SD) [number/time]	4.0 (2.0)	4.1 (1.7)	3.9 (1.9)	0.730	0.671	0.589

SD, Standard Deviation; number, total number of spindles; time, total minutes of Stage II–IV; NREM, Non-REM Stage II–IV; *: P < 0.0167.

**FIGURE 3:**
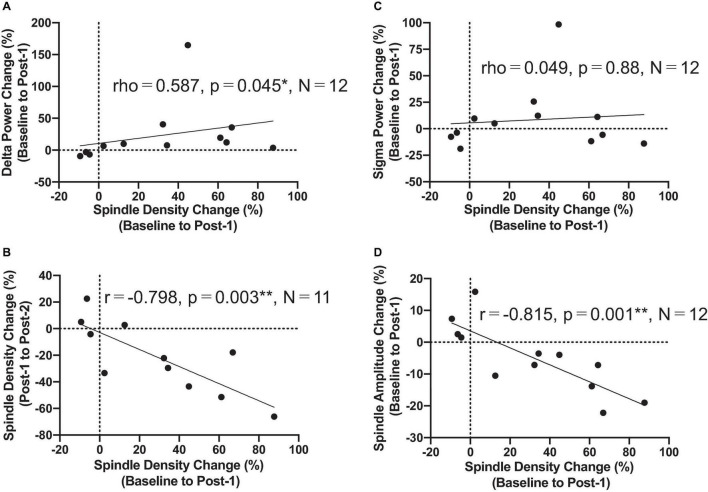
Correlation findings. **(A)** Spindle density change from Baseline to Post-1 was positively correlated with the delta power change during the same period (rho = 0.587, *P* = 0.045, *N* = 12). **(B)** Spindle density change from Baseline to Post-1 was negatively correlated with the spindle density change from Post-1 to Post-2 (*r* = -0.798, *P* = 0.003, *N* = 11). **(C)** Spindle density change from Baseline to Post-1 was not correlated with sigma power change during the same period. **(D)** Spindle density change from Baseline to Post-1 was negatively correlated with the spindle amplitude change during the same period (*r* = -0.815, *P* = 0.001, *N* = 12). *: *P* < 0.05; **: *P* < 0.01.

The most important electrophysiological correlational finding from the present study was the positive correlation between the spindle density increase and the previously reported delta power increase of SWA at the F3 electrode between Baseline and Post-1 (ρ = 0.587, *p* = 0.045, *N* = 12) (Figure 3A). This finding suggests that the two rTMS-associated EEG changes during NREM stages may be concomitant within the thalamocortical network. Additionally, the frontal spindle density increase at F3 between Baseline and Post-1 was inversely correlated with its decrease between Post-1 and Post-2 (*r* = 0.798, *p* = 0.003, *N* = 11) (Figure 3B). This change may result from a homeostatic regulation of sleep spindle activity. Although the sleep spindle density was cross-sectionally correlated with the sigma power during NREM period as in the section “Materials and methods,” their longitudinal changes between Baseline and Post-1 were not correlated at F3 (*r* = 0.095, *p* = 0.77, *N* = 12) and at other electrode sites.

To interpret the background underlying the discrepancy between the increased spindle density and unchanged sigma power density at Post-1, we measured the amplitude and duration of each spindle waveform at F3 electrode. As summarized in Table 3, the absolute number of sleep spindles at F3 increased at Post-1 and then decreased to the baseline level at Post-2, which is a basis of the observed changes of the spindle density. Notably, the mean amplitude of each spindle showed a trend-level (*p* = 0.085) decrease at Post-1, whereas the mean duration did not change over time (*p* = 0.77). Also, the sigma power of each spindle showed a trend-level (*p* = 0.084) decrease at Post-1, whereas the sigma power density did not significantly change over time as we have previously reported (Saeki et al., 2013). In addition, change rates of spindle density and spindle amplitude between Baseline and Post-1 are highly negatively correlated (*r* = −0.815, *p* = 0.001, *n* = 12) (Figure 3D). Given these findings, the discrepancy between the increased spindle density and unchanged sigma power density at F3 is potentially attributed to the decreasing trend in the amplitude of each spindle.

**TABLE 3:** Spindle density and morphology at F3.

	Baseline	Post-1	Post-2	*P*-value (paired *T* tests)		
				Baseline vs. Post-1	Baseline vs. Post-2	Post-1 vs. Post-2
F3 spindle density (SD) [number/time]	3.53 (1.7)	4.76 (2.5)	3.52 (2.2)	0.008**	0.961	0.044*
F3 spindle duration (SD) [seconds/number]	1.06 (0.1)	1.07 (0.1)	1.07 (0.1)	0.767	0.857	0.936
F3 spindle amplitude (SD) [μV/number]	6.63 (1.8)	6.17 (1.2)	6.68 (1.6)	0.085	0.792	0.131
F3 sigma power during NREM (SD) [μV^2/^number]	3.84 (1.5)	3.19 (2.3)	4.28 (2.8)	0.084	0.477	0.177
F3 sigma power during NREM (SD) [μV^2/^time]	11.59 (4.8)	10.92 (4.1)	10.65 (4.2)	0.249	0.412	0.839

SD, Standard Deviation; number, total number of spindles; time, total minutes of Stage II–IV; NREM, Non-REM Stage II–IV; *: P < 0.05; **: P < 0.01.

Clinical and cognitive correlation analyses with the spindle density change at F3 showed no significant correlation, even with an uncorrected alpha level. Though descriptive data of the clinical and cognitive measures were described in detail elsewhere (Saeki et al., 2013), it was briefly noted here that depression symptoms evaluated by HAM-D and BDI, daytime sleepiness by KSS, and executive function by WCST showed significant improvement from Baseline to Post-2.

## Discussion

The present study showed that the increase of the sleep spindle density during natural sleep was highly localized to the F3 electrode, following five sessions of high-frequency rTMS delivered to the left DLPFC of major depression patients. Moreover, this local increase of spindle density at F3 was significantly associated with the previously reported power increase of SWA localized also at F3 in major depression patients. By contrast to the first half period of rTMS sessions, the locally enhanced spindle density at F3 decreased to reach the baseline level during the last series of rTMS sessions. To our knowledge, this is the first study showing a modulation of sleep spindle following rTMS.

Given these findings, it is reasonable to suggest that the localized increases of the sleep spindle density and SWA power could be derived from a common rTMS-induced neuromodulation of the thalamocortical network activity during NREM sleep. The high-frequency rTMS-induced action potentials within cortical pyramidal cells of DLPFC could stimulate not only thalamocortical neurons but also the reticular nucleus of the thalamus, which serve as delta and spindle oscillators, respectively (Gross et al., 2007). It should be noted here that all rTMS sessions were carried out in the morning, to explore long-lasting cumulative effects on PSG data during night rather than the acute after effects of the last rTMS session, which can last for about half an hour (Peinemann et al., 2004).

In our previous work (Saeki et al., 2013), the sigma band power during NREM stages II–IV, which represents an aspect of the sleep spindle activity, was not significantly changed following rTMS sessions in contrast to the localized enhancement of SWA power. The sleep spindle density is likely distinct from a sigma band power, reflecting different aspects of sleep spindle activity. Although both measures were significantly correlated in the cross-sectional dataset, their longitudinal changes did not correlate. The observed discrepancy in the rTMS-induced changes between the spindle density and sigma power density seems attributable to the decreasing trend of amplitude of each spindle waveform during the first half period. Along the lines of this discussion, it can be speculated that the sleep spindle density may be a more sensitive measure to assess rTMS-induced neuromodulation than a sigma power density during NREM stages II–IV.

It should be noted that the observed rise and fall phenomenon of sleep spindle density during a series of rTMS sessions is quite similar for the averaged SWA power, as previously reported (Saeki et al., 2013). Both NREM sleep-related phenomena were localized to the stimulation site of rTMS, and may commonly result from rTMS-induced facilitatory neuroplasticity and subsequent homeostatic downregulation of the locally enhanced thalamocortical synchronization during NREM sleep. Such intrinsic downregulation of NREM sleep may occur within 2 weeks, even during high-frequency rTMS sessions, which should be considered when evaluating direct effects of rTMS on NREM sleep-related phenomena. For instance, a previous study (Pellicciari et al., 2013) failed to detect NREM sleep changes with a 2-week interval of PSG assessments. Accordingly, it could be concluded that an initial follow-up assessment of local NREM sleep activity needs to be scheduled within a week or so after the onset of an rTMS intervention.

With regard to the clinical and cognitive correlation analyses, our basic assumption was that potential rTMS-associated physiological changes of sleep spindle activity can result in clinical or cognitive improvements, especially for executive function, insomnia, and excessive daytime sleepiness. However, exploratory correlation analyses did not suggest any significant association, even with an uncorrected alpha level. Further research with a larger sample size is warranted to determine the clinical/cognitive significance of the rTMS-induced changes of NREM sleep.

The present study has some drawbacks to disclose. First, in an open-label study design, we did not evaluate non-specific effects of a sham stimulation on all-night PSG data, albeit we found a significant change of the sleep spindle density exclusively localized to the stimulation site of the high-frequency rTMS. Second, the present method to identify sleep spindles visually is totally manual and thus could be arbitrary, as compared with an automatic detection algorithm of sleep spindle waveform. Hence, we paid a maximum attention to keeping the single rater (TI) blind to subject profiles particularly for time points of the PSG recording such as Baseline, Post-1 and Post-2. Third, concomitant medication might have impacted on all-night PSG data. As individual medication was fixed throughout the study period, it is less likely that medication could affect longitudinal changes of PSG data. Finally, in this study design, we employed a one-week interval of PSG measurements to avoid excessive burden on depressed patients. However, more frequent PSG measurements enable us to reveal more exact trajectories of the spindle density and associated sigma power during a series of rTMS sessions.

In conclusion, the present study showed a locally and transiently enhanced density of the frontal sleep spindle activity in patients with major depressive episodes, possibly induced by a series of high-frequency rTMS sessions over the left DLPFC. The observed local facilitation of frontal spindle density was transient and downregulated to the baseline level during the last half period of the consecutive 10 rTMS sessions, potentially due to an intrinsic homeostatic regulatory system. Such rise and fall phenomenon of sleep spindle activity is very similar to that observed in SWA and both may be complementary to each other within a framework of thalamocortical synchronization during NREM sleep. Although the clinical/cognitive association with the rTMS-induced changes of sleep spindle density awaits further investigation, the present findings shed light on the neuromodulatory impact of diurnal rTMS sessions on the nocturnal sleep spindle activity.

## Data Availability Statement

The data analyzed in this study is subject to the following licenses/restrictions: Kanagawa Psychiatric Center’s restrictions. Requests to access these datasets should be directed to MN motoaki@motoaki.com.

## Ethics Statement

The studies involving human participants were reviewed and approved by Ethics Committee of the Kanagawa Psychiatric Center (KPC201107). The patients/participants provided their written informed consent to participate in this study.

## Author Contributions

TI, TS, TY, and MN contributed to conception and design of the study. TI analyzed the sleep EEG data using a software developed by NH. TI, MS, and MN performed the statistical analysis. TI and MN wrote the first draft of the manuscript. TI, NH, and MN wrote sections of the manuscript. All authors contributed to manuscript revision, read, and approved the submitted version.

## Conflict of Interest

The authors declare that the research was conducted in the absence of any commercial or financial relationships that could be construed as a potential conflict of interest.

## Publisher’s Note

All claims expressed in this article are solely those of the authors and do not necessarily represent those of their affiliated organizations, or those of the publisher, the editors and the reviewers. Any product that may be evaluated in this article, or claim that may be made by its manufacturer, is not guaranteed or endorsed by the publisher.

## Supplementary Material

Supplementary Figure 1 Spindle morphology and sigma power changes at F3 over time. **(A)** Spindle duration changes from Baseline to Post-2. **(B)** Spindle amplitude changes from Baseline to Post-2. There was a trend-level decrease (*P* = 0.085) between Baseline and Post-1. **(C)** Sigma power per each spindle changes from Baseline to Post-2. There was a trend-level decrease (*P* = 0.084) between Baseline and Post-1. **(D)** Sigma power per minute changes from Baseline to Post-2.
