# Supplementary figures and images for: Local and Transient Changes of Sleep Spindle Density During Series of Prefrontal Repetitive Transcranial Magnetic Stimulation in Patients With a Major Depressive Episode
Source: Front Hum Neurosci. 2022 Jan 6;15:738605. doi: 10.3389/fnhum.2021.738605 (PMC8770927; doi:10.3389/fnhum.2021.738605)

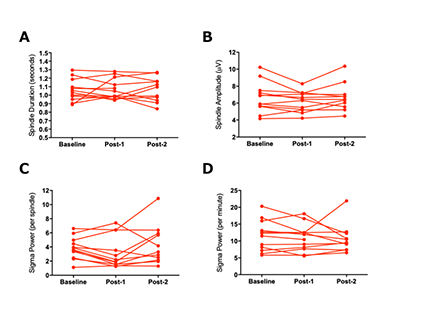

Supplement: Supplementary Figure 1 — Spindle morphology and sigma power changes at F3 over time. (A) Spindle duration changes from Baseline to Post-2. (B) Spindle amplitude changes from Baseline to Post-2. There was a trend-level decrease (P = 0.085) between Baseline and Post-1. (C) Sigma power per each spindle changes from Baseline to Post-2. There was a trend-level decrease (P = 0.084) between Baseline and Post-1. (D) Sigma power per minute changes from Baseline to Post-2. [file Image_1.tif]
